# Supplementary material for: Evaluation of fendiline treatment in VP40 system with nucleation-elongation process: a computational model of Ebola virus matrix protein assembly
Source: Microbiol Spectr. 2024 Feb 26;12(4):e03098-23. doi: 10.1128/spectrum.03098-23 (PMC10986538; doi:10.1128/spectrum.03098-23)
Supplement: Supplemental material — Figures S1 to S6; Tables S2 and S4 to S10. [file spectrum.03098-23-s0001.pdf]

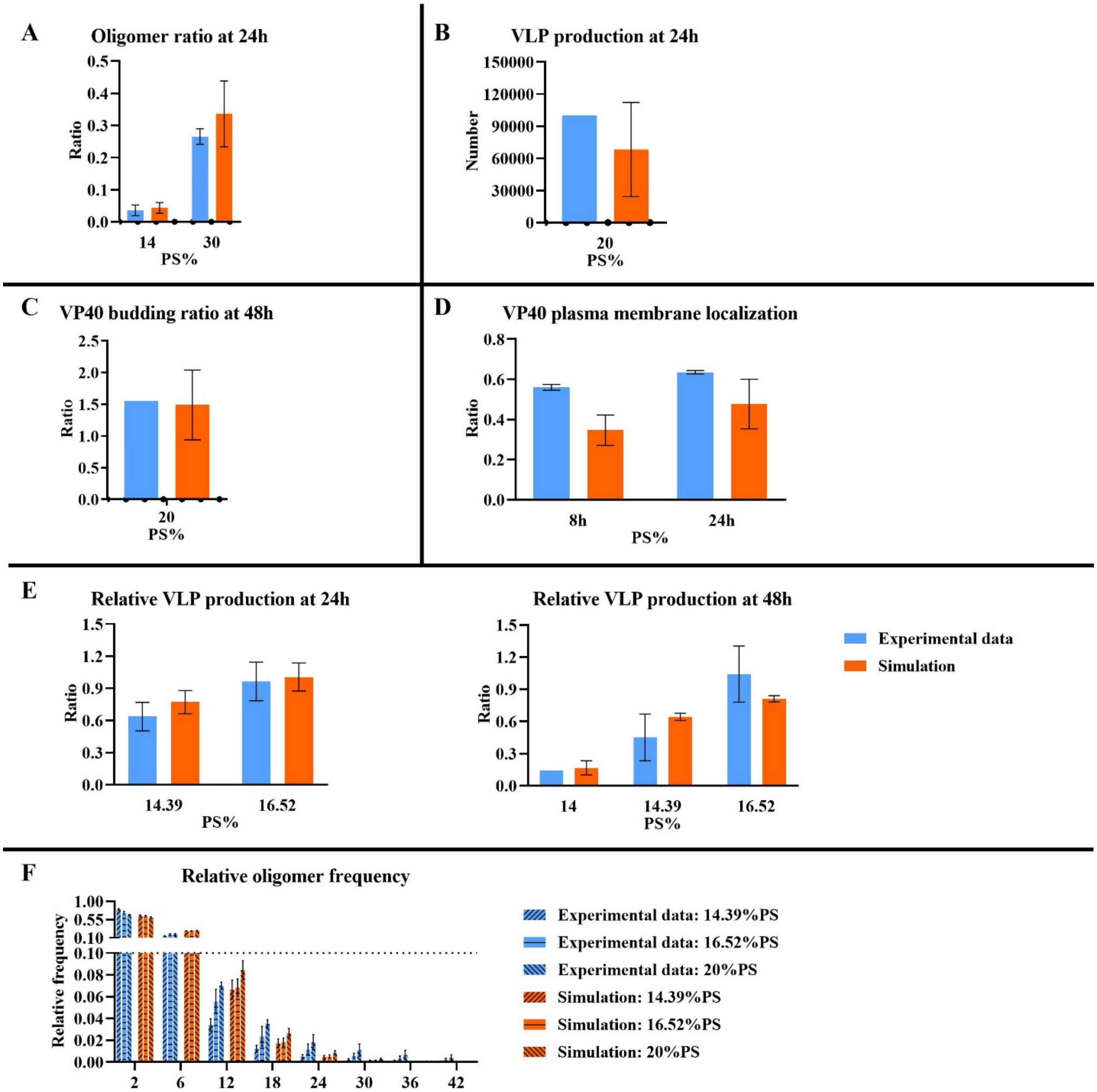

Figure S1. Simulation from the 'As2' model. (A) Oligomer ratio at 24 h. (B) VLP production at 24 h. (C) VP40 budding ratio at 48 h. (D) VP40 plasma membrane localization. (E) Relative VLP production. (F) Relative oligomer frequency. Both the deceasing trend of relative frequency from membrane VP40 dimer to 42mer, and the increasing trend in higher oligomers under higher PS level are reproduced. The three bars in each of the sub-column are 14.39%, 16,52%, 20% PS from left to right separately. Error bars indicate SEM from top 5 fits.

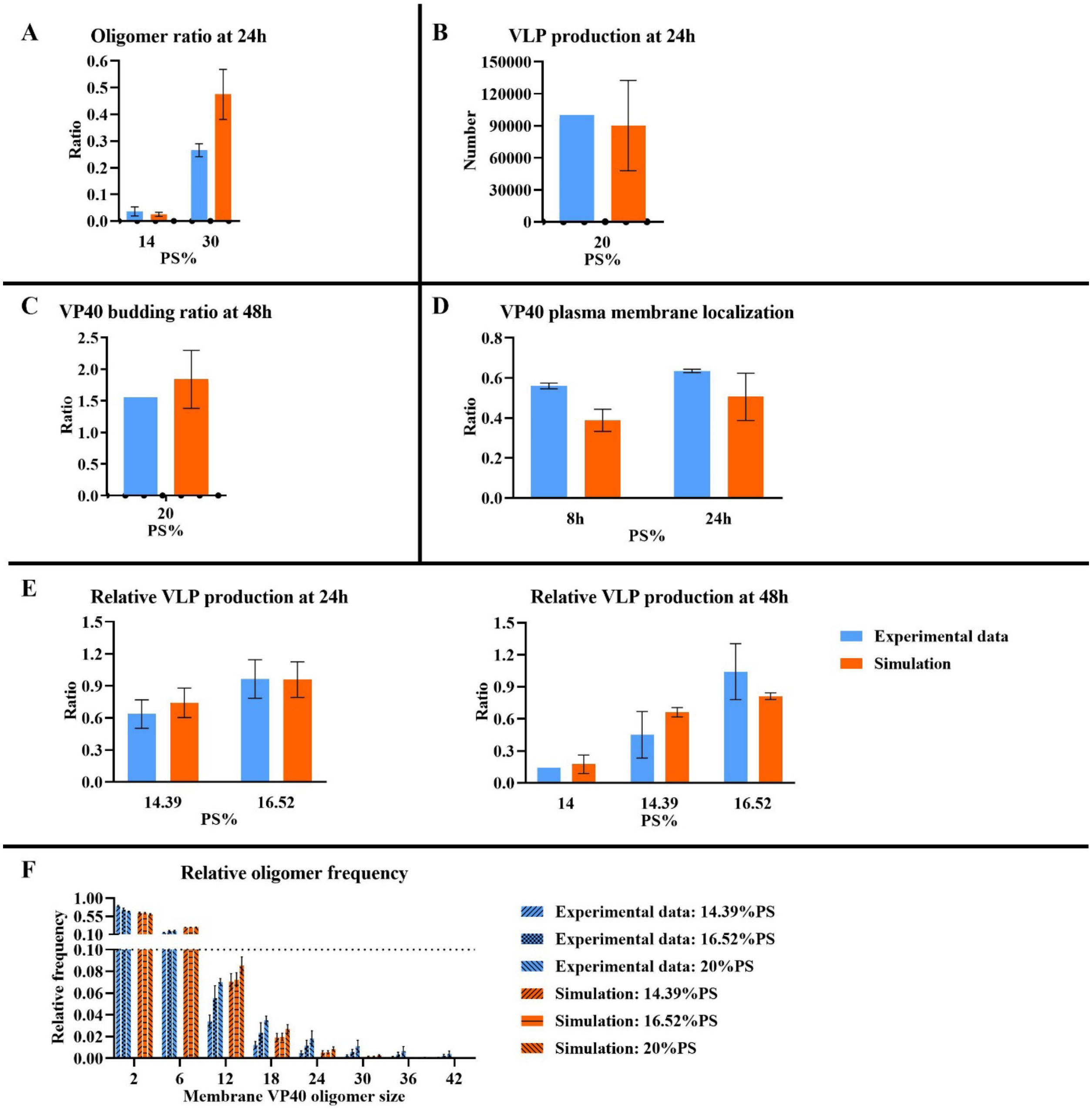

Figure S2. Simulation from the 'As3' model. (A) Oligomer ratio at 24 h. (B) VLP production at 24 h. (C) VP40 budding ratio at 48 h. (D) VP40 plasma membrane localization. (E) Relative VLP production. (F) Relative oligomer frequency. Both the deceasing trend of relative frequency from membrane VP40 dimer to 42mer, and the increasing trend in higher oligomers under higher PS level are reproduced. The three bars in each of the sub-column are 14.39%, 16,52%, 20% PS from left to right separately. Error bars indicate SEM from top 5 fits.

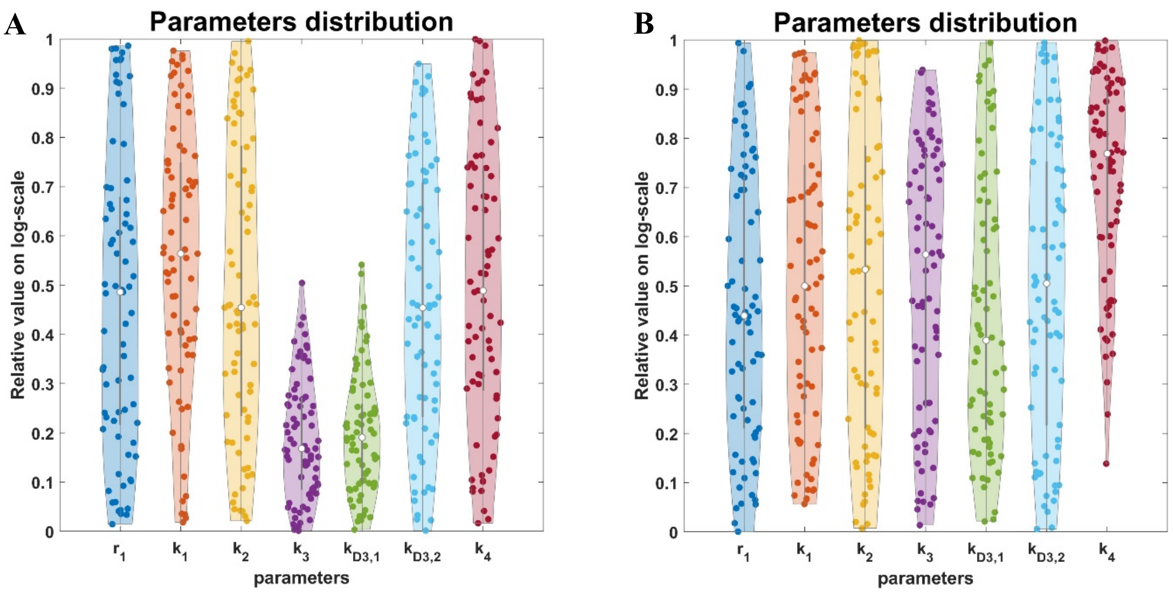

Figure S3. Parameter distribution of groups with fendiline treatment induced VLP production in late and early budding dynamic groups. (A) Fendiline-induced VLP increase groups in late VLP production type have typical low k_3 and $k_{3,1}$'. (B) Other Fendiline-induced VLP increase groups are more related to high k_4. Values are normalized to the sampling range of parameters. The Y axis range shows the relative value of each parameter in their LHS. 0 indicates lower bound and 1 indicates upper bound.

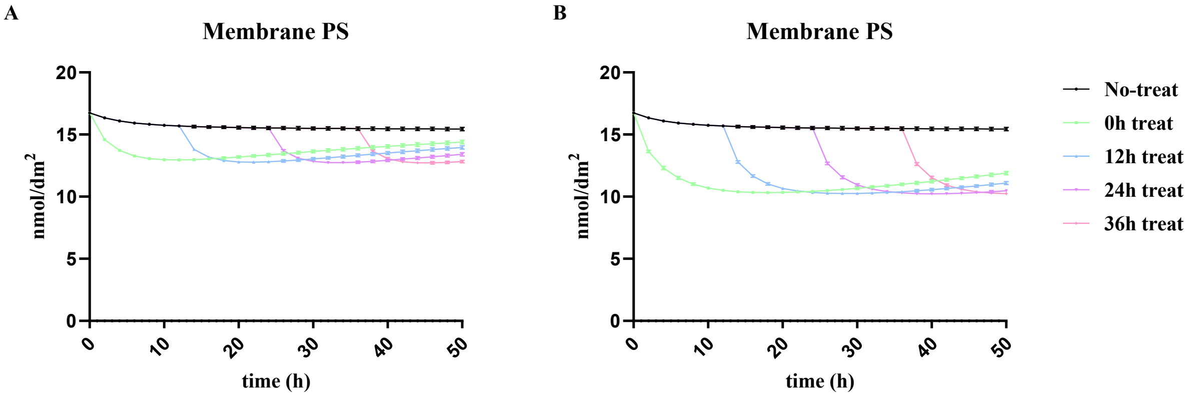

Figure S4. PS dynamic of one-dose fendiline simulation. (A) 2μM of fendiline applied at 0, 12, 24 and 36 h post infection. (B) 10μM of fendiline applied at 0, 12, 24 and 36 h post infection. Error bars indicate the SEM. The figure is plotted based on every two hours.

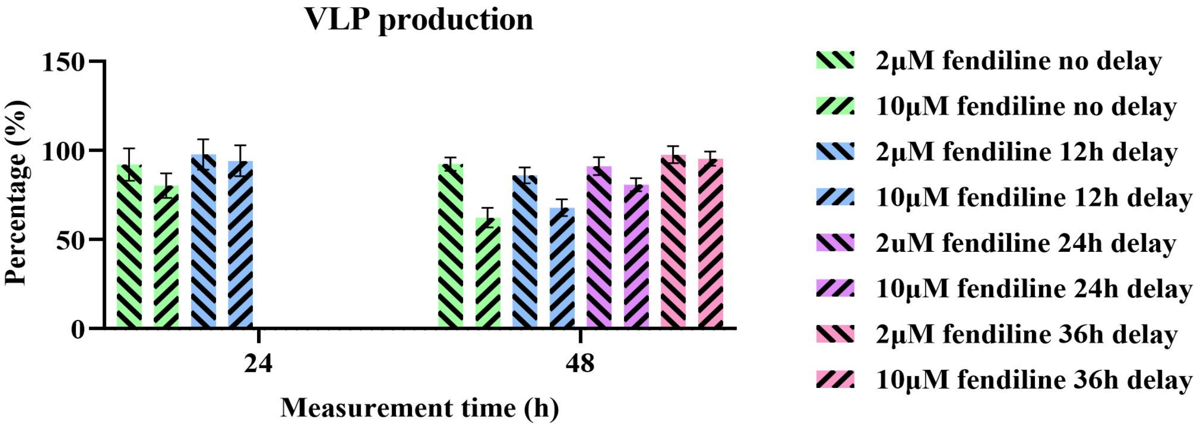

Figure S5. Difference in VLP production between 2 μM and 10 μM applied at different time experimentally. Difference in VLP production between 2 μM and 10 μM fendiline application decreases with later application time. The difference in VLP measurement is not significant at 24 h when fendiline is applied at 0 or 12 hours, and at 48 h when fendiline is applied at 24 or 36 hours (Table S10). fendiline Error bars indicate the SEM.

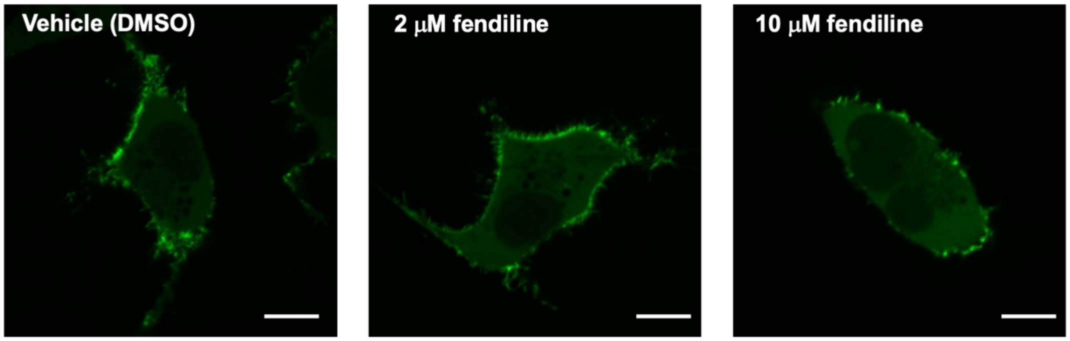

Figure S6. Representative confocal images of HEK293 cells expressing EGFP-VP40 with vehicle (DMSO) or fendiline treatment. HEK293 cells post-VP40 transfection were treated with either vehicle (DMSO) or fendiline (at varying concentrations for analysis at different time points 24 or 48 hours). Confocal imaging was performed (48 hours as in Fig. S5) and image analysis (plasma membrane localization pre-VLP formation) was performed by counting pre-VLPs at the plasma membrane per cell slice by moving the Z plane of the image of and down. The number of pre-VLPs were counted per imaging frame for an equal number of VP40 expressing cells over the course of three independent experiments and normalized to WT VP40 production with vehicle treatment. Scale bar = 10 mm.

Table S1. Model parameters.
See "Supporting_information_model_parameters.xlsx"

Table S2. Statistical analysis for parameter distribution in fendiline-induced VLP production simulation.

P-values		Type of fendiline-induced VLP production dynamic		
		All	Late VLP production type	Early VLP production type
Parameters	r_1	0.1859	0.626	0.1791
	k_1	0.4885	0.1769	0.9033
	k_2'	0.5493	0.4801	0.1839
	k_3	<0.0001	<0.0001	0.7243
	$K_{D3,1}$	<0.0001	<0.0001	0.0456
	$K_{D3,2}$	0.9606	0.6493	0.9054
	k_4	0.0005	0.9983	<0.0001

*Two-tailed unpaired t test was performed.

Table S3. Efficiency of fendiline application at different time: Experiment.
See "Supporting_information_Efficiency_of_fendiline_application_at_different_time.xlsx"

Table S4. Statistical analysis for impact of fendiline application time on VLP production (Simulation).

VLP production %	Predicted time point	group	p-value	
			2μM fendiline	10μM fendiline
Constant fendiline concentration	24h	0h VS 12h	<0.0001	<0.0001
	48h	0h VS 12h	<0.0001	<0.0001
		12h VS 24h	<0.0001	<0.0001
		24h VS 36h	<0.0001	<0.0001
Dynamic fendiline concentration	24h	0h VS 12h	0.6851	<0.0001
	48h	0h VS 12h	<0.0001	0.0002
		12h VS 24h	0.9972	<0.0001
		24h VS 36h	<0.0001	<0.0001

*Two-tailed paired t-test was performed at 24h predicting time.
*Paired one-way ANOVA multiple comparasion was performed at 48h predicting time.

Table S5. Statistical analysis for impact of fendiline application time on treatment efficiency per hour (Simulation).

Treatment efficiency per hour	Predicted time point	group	p-value	
			2μM fendiline	10μM fendiline
Constant fendiline concentration	24h	0h VS 12h	<0.0001	<0.0001
	48h	0h VS 12h	<0.0001	<0.0001
		12h VS 24h	<0.0001	<0.0001
		24h VS 36h	<0.0001	0.0212
Dynamic fendiline concentration	24h	0h VS 12h	<0.0001	<0.0001
	48h	0h VS 12h	<0.0001	<0.0001
		12h VS 24h	<0.0001	<0.0001
		24h VS 36h	<0.0001	<0.0001

*Two-tailed paired t-test was performed at 24h predicting time.
*Paired one-way ANOVA multiple comparasion was performed at 48h predicting time.

Table S6. Statistical analysis for impact of fendiline concentration on VLP production at different application time (Experiment).

p-value		Fendiline application time			
		0h	12h	24h	36h
Measurement time	24h	0.3595	0.7821	N/A	N/A
	48h	0.0105	0.0483	0.1685	0.7452

*Two-tailed unpaired t test was performed.

Table S7. PS inhibition experimental data

Relative PS production (r_2/r_{2WT})	Relative PS level (PS/20)
3	0
0.660079	1.033333
0.544001	1.066667
0.339101	1.133333
0.189839	1.2
0.132573	1.266667
0.074285	1.333333

Table S8. Fendiline concentration experimental data

$Conc_{Fendiline}$ (uM)	$C_{Fendiline}/C_{WT}$
1	0.825
5	0.72
10	0.602

Table S9. Weight of cost calculation

Data	w
VLP production number	0.25
VP40 oligomer ratio	1
Relative oligomer frequency	1
Relative VLP production	1
VP40 budding ratio	1
VP40 plasma membrane localization	1

Table S10. Statistical analysis for membrane dimer concentration

P-value	24h	48h
No treat VS 2 μM fendiline	<0.0001	<0.0001
2 μM fendiline VS 4 μM fendiline	<0.0001	<0.0001
4 μM fendiline VS 6 μM fendiline	<0.0001	<0.0001
6 μM fendiline VS 8 μM fendiline	<0.0001	<0.0001
8 μM fendiline VS 10 μM fendiline	0.1210	0.0057

*Paired one-way ANOVA multiple comparasion was performed.
